# Review of the environmental and health risks of hydraulic fracturing fluids

**DOI:** 10.1016/j.heliyon.2024.e40883

**Published:** 2024-12-03

**Authors:** Sara Makki, Elsa Maalouf, Alissar Yehya

**Affiliations:** a Baha and Walid Bassatne Department of Chemical Engineering and Advanced Energy, Maroun Semaan Faculty of Engineering and Architecture, American University of Beirut, Lebanon; b Department of Civil and Environmental Engineering, Maroun Semaan Faculty of Engineering and Architecture, American University of Beirut, Lebanon; c Harvard John A. Paulson School of Engineering and Applied Sciences, Harvard University, Cambridge, USA

**Keywords:** Hydraulic fracturing, Fracturing fluid, Recovery, Environment, Risk, Safety

## Abstract

The composition of hydraulic fracturing (HF) fluid poses risks to human health and the environment by impacting drinking water sources. Fracturing fluid recovery rate is highly variable, and the fact that a high percentage of the injected HF fluid is not produced back to the surface in some areas raises questions about its fate and possible migration into aquifers. In this paper, the composition of the HF fluid and related toxicity are described, along with insights about the environmental impact linked with HF fluid, synthesized spill data, main factors affecting the flow-back ratio, and induced seismicity related to HF activities. The environmental and health hazards posed by HF fluid are concerning due to the high concentration of toxic chemicals, the limited data on toxicity, the high probability of spills, and the reported cases of aquifer contamination. Furthermore, low load recovery values (10%–50 %) suggest that a significant volume of fracturing fluids may remain in the subsurface, thereby potentially increasing the likelihood of fluid migration towards drinking water sources under certain conditions. Hence, the fate of HF fluid is explained by establishing correlations between fluid flow (i.e., flow-back and migration to the subsurface) and different operation and formation parameters. For example, a negative correlation was detected between HF fluid recovery and shut-in period, fracture network complexity, and induced seismicity, while a positive correlation was observed between HF fluid migration speed and permeable pathways. Moreover, it is shown that the main handicap in assessing related risks is the scarcity of disclosure and monitoring data. Consequently, future work must focus on imposing strict disclosure and incident-reporting regulations, and more publications should be dedicated to inspecting the composition and impact of HF fluid.

## 1 Introduction

The use of unconventional oil and gas production technologies is increasing due to the continuous increase in energy demand [1]. Accordingly, the exploitation of unconventional reservoirs through hydraulic fracturing is an inevitable choice in the industry [2]. Hydraulic fracturing (HF) operations inject large and highly pressurized volumes of fracturing fluid into tight shale formations to fracture the rock and allow the extraction of trapped oil and gas through the created flow pathways [1,3,4]. The most widely used type of fracturing fluid is slick-water [5], which is a water-based fluid that contains a fraction of added chemicals (e.g., friction reducers) and proppants [6,7]. Flowback and produced water returning to the surface during production consist of the fraction of the injected fracturing fluid that is recovered and the brine, respectively [8,9]. Reported fracturing fluid flow-back recovery values vary significantly across different formations, reflecting that a substantial volume of fluid typically remains in the subsurface [3]. For example, recovery rates for the Marcellus shale are reported at 7.8 % [11], for the Qusaiba formation at 9.6 % [12], for the Montney formation at 36 % [13], and for the Haynesville formation at 5 % [9]. These examples highlight the generally low flow-back recovery rates across formations, which contributes to notable fluid retention in the subsurface.

Meanwhile, environmentalists are constantly warning on the potential risks associated with the migration of the remaining fraction of this fluid (>70 %) which imbibed into the shale formation. Particularly, there are concerns on possible groundwater contamination because the additives present in the fracturing fluid might find their way to water aquifers [2,4,14,15]. Toxicity information available about fracturing fluid chemicals show that being exposed to fracturing fluid poses serious health hazards on humans [[15], [16], [17], [18]]. Public pressure concerning this issue is constantly intensifying because toxicity data is limited and disclosures of fracturing fluid are questionable. Previously, voluntary disclosures were minimal [19], and mandatory disclosure regulations used to have shortcomings concerning the chemicals' identification requirements, imposed penalties in case of insufficient reporting, and lenient timelines of reporting [20]. A recent study by Bonetti et al. [19] showed that revisiting and tightening disclosure mandates led to a decrease in the quantity of toxic chemicals used. Assessing the environmental impact of fracturing fluid, monitoring its fate, and tracking the diffusion of the fluid's constituents post injection are necessary to eliminate possible hazards.

Unfortunately, the fate of the injected fluid in the subsurface is not clearly understood [[3], [10]]. So, investigating the fate of the un-recovered fraction of the fluid requires a thorough understanding of the fluid transport in the subsurface post injection. The literature highlights the issues of the un-recovered fracturing fluid by addressing three main topics:

(i) Upward fluid migration into drinking water sources [[21], [22], [23], [24], [25], [26], [27], [28]],
(ii) fluid retention in complex fractured systems created due to hydraulic fracturing [5,[29], [30], [31], [32]], and
(iii) the effect of operational parameters (e.g. shut-in period) on fluid recovery [21,[33], [34], [35], [36], [37], [38]].

Accordingly, as this review paper demonstrates, the key parameters controlling the flow-back in hydraulic fracturing are primarily related to formation and operational properties (e.g., permeable pathway properties, shut-in time, and fracture network complexity and properties).

Furthermore, instances where induced seismicity was correlated with hydraulic fracturing are presented and provide further evidence that fracturing fluids are diffusing and migrating to permeable underground formations. Fluid diffusion has been identified as the primary trigger behind recorded earthquakes in western Canada [[39], [40], [41], [42]] and the Sichuan Basin in China [43,44]. Hence, low flow back recovery may be attributed to underground fluid diffusion in addition to fluid imbibition into the matrix, retention within the fracture systems, and potential upward migration.

Additionally, although hydraulic fracturing is widely recognized for its use in the oil and gas industry, its application has expanded into sustainability-focused projects such as carbon capture and storage (CCS) and enhanced geothermal systems (EGS). In these areas, hydraulic fracturing is used to improve permeability, create heat reservoirs in EGS, and enhance CO₂ injectivity in storage reservoirs. Although the types and concentrations of chemicals used in fracturing fluids for CCS and EGS are typically simpler and lower than those in oil and gas applications, there remain critical environmental risks—such as induced seismicity, fluid migration, and CO₂ leakage—that are important to consider. These shared concerns highlight the broader relevance of hydraulic fracturing fate studies, as they illuminate fluid retention and migration behaviors that could affect subsurface and surface water resources across applications. By evaluating hydraulic fracturing's impacts in CCS and EGS alongside traditional oil and gas contexts, we gain a more comprehensive understanding of how this technology can support, rather than undermine, sustainability goals. Addressing these risks proactively is essential to ensuring that hydraulic fracturing contributes positively to energy transition efforts and environmental protection measures [[45], [46], [47], [48], [49], [50], [51], [52]].

This review is essential for addressing a significant gap in understanding the environmental impacts of fracturing fluid and has two primary objectives: first, to compile data on the composition of fracking fluid and the associated hazards which highlight the environmental concerns rising from the uncertainty surrounding the fate of the fracturing fluid; and second, to establish correlations between the environmental and geologic observations, such as water contamination, induced seismicity, subsurface complexity and the recovery rate.

## 2 Methodology

Peer reviewed articles from scientific journals, Environmental Protection Agency (EPA) reports, and conferences papers were analyzed and discussed in order to describe the fate, transport and recovery of fracturing fluids, in addition to their environmental and health impacts. The 100+ papers referenced in this review included both numerical simulations and case studies. Accordingly, the articles referenced are classified into three categories representing the main areas of the study, which are (1) environmental impact of HF fluid and associated risks; (2) fate of HF fluid; and (3) induced seismicity triggered by HF fluid diffusion and its correlation to flow back.

First, HF fluid composition and toxicity are illustrated to emphasize the criticalness of surface and subsurface risks associated with the transport and migration of HF fluid. Next, the fate of HF fluid was examined due to the subsurface threats posed by its migration, particularly the retention of a significant fraction of HF fluid in the subsurface post-production. This analysis involved exploring the potential reasons behind the low flowback recovery values and establishing correlations between HF fluid recovery and the various factors affecting it. Finally, a correlation between induced seismicity and load recovery is demonstrated by highlighting the spatial and temporal link between seismic activity and hydraulic fracturing operations in which HF fluid migrates to critically stressed faults. Moreover, this research identifies the limitations and gaps in the literature, which are related to the scarcity of data. Disclosure data about the composition of the fracturing fluid is limited [19,20], and subsurface monitoring data utilized to characterize fluid migration are rare in comparison to the large number of hydraulically fractured wells [53]. Such limitations impede groundwater contamination investigations and restrict the publishing of relevant studies, leading to shortcomings in irrefutable documentation of drinking water contamination by fracturing fluid [54].

Certainly, as this is a controversial topic in the industry, the papers were reviewed from a scientific point of view without any bias. Furthermore, the studies were selected from various sources and geographic locations to avoid biases and ensure objectivity in the analysis.

## 3 Environmental impact of fracturing fluids

### 3.1 Fracturing fluid composition

Fracturing fluid composition, required for the success of the fracturing operations, consists of a base fluid (e.g. water, acid, oil), proppants, and chemical additives such as gelling agents, crosslinkers, corrosion inhibitors, and friction reducers. Since the introduction of hydraulic fracturing, there has been a continuous development in fracturing fluid composition, shifting from a hydrocarbon to an aqueous basis [55]. Among the various base fluids and their corresponding fluid types (see Table 1), the fluid composition selection is based on the formation targeted and fracturing job results anticipated [[55], [56], [57], [58]].

**Table 1:** Fluid types employed for the different types of fracturing fluid base [56].

Base Fluid	Fluid Type
Water	Linear gel
	Crosslinked gel
	Slickwater
	Viscoelastic surfactant gel
Oil	Linear gel
	Crosslinked gel
	Emulsion
Acid	Linear gel
	Crosslinked gel
	Emulsion
Alcohol	Methanol and water mixture
	100 % Methanol
Foam	Water-based
	Acid-based
	Alcohol-based
	CO_2_-based
Emulsion	Water-oil emulsion
	CO_2_-methanol

**Fig. 1:**
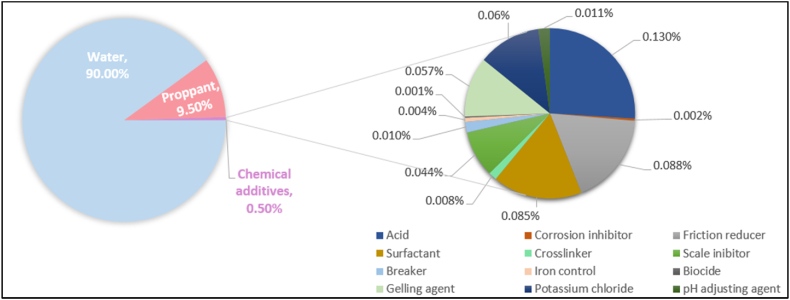
Water-based fracturing fluid composition (reproduced from Ref. [58]).

Recent literature indicates that the most commonly used fracturing fluid is water-based, with a typical description as detailed in Fig. 1 [2,14,56,[58], [59], [60], [61], [62], [63]]. However, specific regions, such as California, often employ alternative fluids like acid-based fracturing fluids due to unique geological and resource characteristics, highlighting how local conditions can influence fracturing fluid selection.

Operators state that only a small percentage of the fluid is occupied by chemicals and about 99 % is occupied by the water and proppants. Nevertheless, this does not negate the need to counterattack the negative consequences resulting from the usage of fracturing fluid [14,17,55] and the fact that hydraulic fracturing treatments require large volumes of fluid and thus a large volume of chemicals is used regardless of their percentage in the composition [59,60]. Accordingly, regulatory authorities demand operators to provide a disclosure of the chemical additives present in the hydraulic fracturing fluid [57]. For example, this data is available for many states in the USA [19] and for Alberta and British Columbia, which are the provinces with the highest fracturing activity in Canada [64].

### 3.2 Toxicity of additives

Chemical additives in the fracturing fluid pose a threat on the environment and human health [14,16,17]. Environmentalists are especially alert regarding the issue of threats posed on groundwater's integrity due to possible contamination by fracturing fluid chemicals, which are labeled as “chemicals of concern”, due to their toxicity (e.g., mammalian, aquatic) and human health related hazards (i.e., effects on nervous system, immune system, respiratory system, reproductive system, and sensory organs) [15,54,[65], [66], [67]]. Nevertheless, severity of these effects varies and is controlled by degree and way of exposure to these chemicals.

**Fig. 2:**
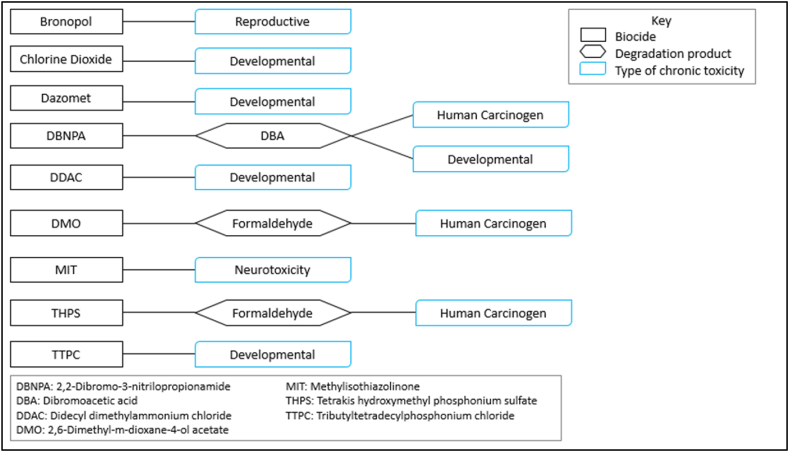
Toxicological data of biocides used in hydraulic fracturing operations [54].

In studies dedicated to evaluating toxicity of additives present in hydraulic fracturing fluids, authors [15,54,65,67] usually follow the same approach; compiling lists of disclosed chemicals, then scanning databases for toxicological data, and possible health effects. Key findings from the studies provide different pieces of information about the type of toxicity associated with the chemicals, toxicity ratings, hazard rankings, and associated health effects. Table 2 shows some of the interesting findings presented in the literature. Furthermore, Kahrilas et al. [54] reviewed the toxicity of biocides used in fracturing fluids, highlighting their potential as serious irritants to sensory organs and their considerable acute toxicity to aquatic life. Data from their study are summarized and illustrated in Fig. 2 to show the types of chronic toxicity associated with certain biocides and their degradation products used in fracturing fluid formulations.

**Table 2:** Summary of toxicity data availability for hydraulic fracturing fluid chemicals as reported in each study, including the types of data presented and the biological systems affected.

Number of chemicals studied	Toxicity data availability	Toxicity data available/most affected systems	Reference
1076	10 %	Chronic oral toxicity values	[68]
36	22 %	Chronic oral toxicity values Note: Additives reported in fracturing fluids in at least 10 % of wells in the U.S.	
600	58.8 %	• >75 % affect sensory organs, respiratory and gastrointestinal systems • 52 % affect nervous system, immune and cardiovascular systems, and kidneys • 37 % affect endocrine system • 25 % cause cancer	[65]
81	69 %	• 16 % low/moderate toxicity • 31 % lacked toxicity data • 53 % considered as non-toxic	[63]
168	N/A	14 % associated with reproductive and developmental toxicity	[69]
320	60 %	Chronic and/or acute hazard screening values	[66]
924	21 %	Reproductive and developmental toxicity reported	[15]
28	82 %	Mammalian and aquatic acute toxicity measurements Note: Additives used in more than 10 % of fracturing treatments in California	[67]
20	45 %	Mammalian and aquatic acute toxicity measurements

Despite extensive data collection and studies demonstrating the toxicity of fracturing fluid constituents, comprehensive toxicity assessment remains challenging. In studies examining these chemicals, toxicity data is often incomplete, with many chemicals lacking toxicity information [15,67,68]. Furthermore, as Caldwell et al. [70] notes, the information disclosed by industries on the chemicals used is still not detailed enough, and this lack of transparency continues to hinder scientists' ability to assess potential chemical interactions in the subsurface and the resulting changes in toxicity.

### 3.3 Associated risks

From an environmental perspective, risks associated with the use of fracturing fluid are numerous. A significant portion of the literature addresses the risk of water bodies contamination, which can be the result of aboveground incidents (e.g., spillage of the fracturing fluid during transport) or the infiltration of the fluid into aquifers through migration into subsurface layers [17,18,53,66,[71], [72], [73], [74], [75], [76], [77], [78], [79]].

One of the pre-requisites required to assess environmental hazards, as per Shah et al. [18], is tracking the motion of the hydraulic fracturing fluid. Similar to Kreipl & Kreipl's [73] classification, they categorized possible risks into two groups: surface risks and downhole risks.

#### 3.3.1 Surface risks

Risks classified as surface risks primarily include fracturing fluid spills that occur during transport and leakage incidents from storage pits [17,77,79,80]. Mrdjen & Lee [74] emphasized the adverse human health effects due to possible contamination of surface water bodies, often utilized as sources of drinking water, by chemicals used in hydraulic fracturing operations [81,82]. This important issue has not been addressed considerably in the literature. Fracturing fluid and flow-back and/or produced water spills and leakage can occur both prior to and during injection (e.g., spills during the transport of fracking water and additives to the site for mixing, and leakage from storage tanks) and throughout production (e.g., accidental spills of produced water on the soils or illegal deliberate releases by some operators) [8,74,83].

**Fig. 3:**
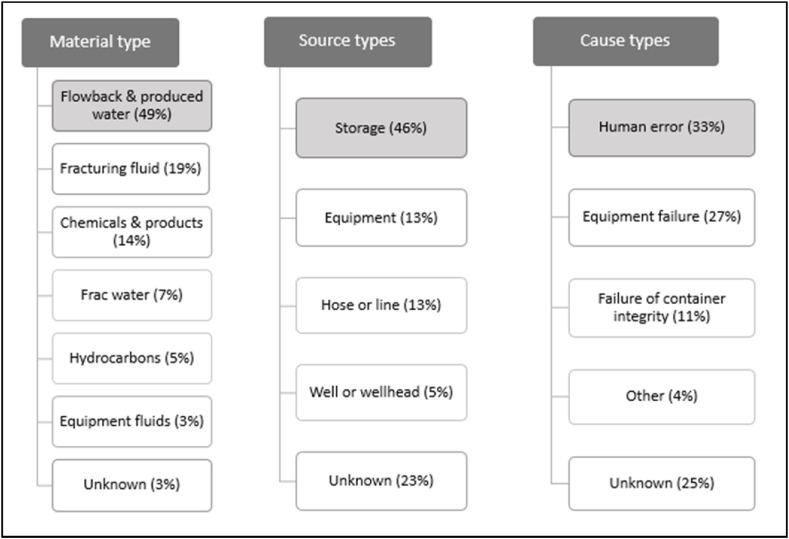
Percent distribution of material related to hydraulic fracturing that are spilled, their source and cause. These values were calculated using the number of spills per material, source, and cause types relative to the total number of spills with data synthesized from the U.S. EPA [76].

Numerous studies have compiled and analyzed spill records [8,54,71,75,76,80,[83], [84], [85]]. Scientists are able to categorize spills linked to hydraulic fracturing by type of material, pathways, sources, causes, and volume spilled [75,76,80]. For example, the EPA report [76] examined data of more than 36,000 spills recorded over a period of 6–7 years in 11 states in the U.S. and found that the most prevalent material types that are spilled are flow-back and produced water (49 % of the total material spilled), the source of these spills are mainly from storage (46 % of the total source type of spills), and the cause is mainly due to human error (33 % of the time) and equipment failures (27 % of the time). A detailed description is synthesized in Fig. 3.

As a demonstration of spill data analysis in states with significant unconventional oil and gas activity, spill rates calculated by Maloney et al. [80] in Colorado, New Mexico, North Dakota, and Pennsylvania are presented in Table 3. Differences in spill rates across states may reflect variability in reporting requirements, which differ in terms of volume thresholds, spill location specifications, and methods of reporting (written or verbal).

**Table 3:** Spill rates calculated from spill data collected between 2005 and 2014 across selected U.S. states [80].

Location	Spill rate (%)	Data timeframe	Data source
State of Colorado	7.1 %	2005–2014∗^a^	Colorado Oil and Gas Conservation Commission
New Mexico	12.1 %		New Mexico Oil Conservation Division
North Dakota	40.7 %		Oilfield Environmental Incident reports
Pennsylvania	17.6 %		Notice of violations (NOV) database

a For Colorado, the data timeframe is from January 1, 2005, to December 31, 2013, due to a significant change in spill reporting starting in 2014. While for the other states, the timeframe is from January 1, 2005, to December 31, 2014.

**Fig. 4:**
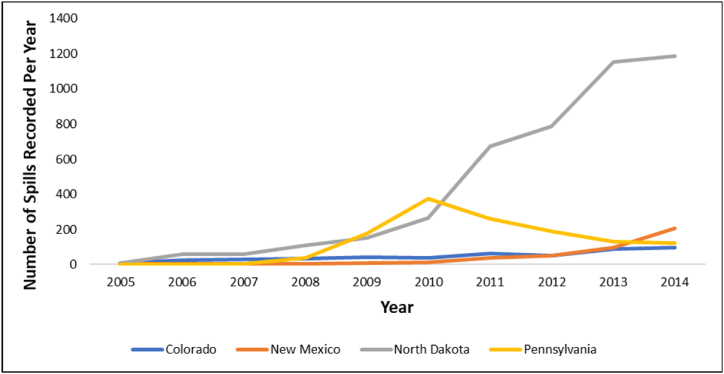
Annual number of reported spills from 2005 to 2014 across four states [75].

To further analyze state-specific trends, we highlight annual variations in reported spill numbers as documented by Patterson et al. [75] (see Fig. 4). Fluctuations within each state, along with an overall upward trend in reported spills, suggest that tighter reporting requirements have increased reported spill counts [75,77]. Notably, North Dakota experienced a marked rise in reported spills post-2010, the year which marks the transition from verbal to written reporting obligations in the state [75]. As for the State of Colorado, regulatory changes in 2008 and 2013, which included lowering the minimum reportable spill volume to 1 barrel in 2013 [77] likely contributed to the consistent rise in number of reported spills.

It must be highlighted that although the spill rates usually reported might be misinterpreted as inconsequential, the volume per spill ranges from small values like 1 Gal to critically high values reaching 1 MGal [71,75,76], thereby amplifying exposure risks. Estimating fracturing fluid spill rates serves as a basis for spill assessment. Spills of all material types, even if non-toxic, must be incorporated into spill probability assessments, since a spill during transport due to a wreck incident has the same likelihood whether the fluid is fracturing fluid or any other chemical; if the content is not safe it will pose a serious environmental hazard. Another important factor in assessment and mitigation practices is monitoring how changes in reporting requirements impact the spill rates recorded, where stricter mandates are leading to an improvement in accuracy, details, and number of reported spills.

#### 3.3.2 Downhole risks

Groundwater is an invaluable and versatile resource; in countries like the United States, reliance on groundwater for purposes such as public use, irrigation, livestock, and human consumption is significantly higher than on surface water sources, mainly because it is less susceptible to pollution compared to surface water [78]. So, it is vital to prevent groundwater contamination.

Nevertheless, the downhole risks associated with hydraulic fracturing present a distinct threat to groundwater quality, as they primarily relate to the possible upward fracturing fluid migration towards drinking water aquifers [14,17,65]. A considerable amount of the literature has discussed the potential pathways, and it was found that, mainly, pathways can be created as a consequence of poor well integrity (e.g., poor cementing) and/or undesired hydraulic communication in the subsurface [2,18,53,66,78,79,[86], [87], [88]].

Researchers identified the most common pathways:

1. Defective casing installments and/or poor cementing jobs.
2. Intersection between created hydraulic fractures and aquifer.
3. Intersection between created hydraulic fractures and pre-existing fractures or faults.
4. Intersection between created hydraulic fractures and adjacent wells (e.g., abandoned wells, offset wells).

Although hydraulic fracturing design and operation plans should be prepared to avoid undesirable fluid leakage, well design and planning has a level of uncertainty. Faulty cementing, leaky casing connections, casing corrosion, and cement sheath degradation are contributors that jeopardize well integrity and promote fluid loss [78,79,87,88]. For example, shallow leaky casings near the aquifers, and annular spaces created by cement sheath failure form channels and conduits for the fracturing fluid to infiltrate into unfavorable zones [78,88]. Also, initiated fractures might extend out of the designated interval and provoke unanticipated communication, especially in the presence of unmapped faults [2,18,53,66,86]. Furthermore, pathways generated due to communication between hydraulic fractures and wells are attributable to well communication/interference. One of the most important types of this interference is fracture communication, known as frac-hits, and it is highly controlled by well spacing, where tighter spacing leads to a higher degree of interference among wells [89]. Simply put, groundwater contamination is possible through these pathways if two conditions are met: presence of a fluid and a driving force for fluid flow [53].

Despite the fact that groundwater contamination by hydraulic fracturing additives has rarely been irrefutably documented in the academic literature and such events are considered to be of relatively low occurrence, there have been reported contamination incidents of drinking water sources by the fracturing fluid and the health implications are alarming. Notable examples of water contamination linked to hydraulic fracturing operations are.

A. Jackson County, West Virginia in 1982: fracturing fluid gel was detected possibly due to a nearby gas wells that could serve as conduits for fluid flow [90].
B. West Divide Creek in Garfield County, Colorado in 2004: BTEX (benzene, toluene, ethylbenzene and xylene) was detected possibly due to natural fractures and faults and poor wellbore integrity (i.e., improper cementing job) that could serve as conduits for fluid flow [91].
C. Pavillion in Fremont County, Wyoming in 2008: Fracturing fluid components possibly due to the short distance between the aquifer and the well [72].

On another note, even though methane detection in drinking water is out of the scope of this paper, the literature is rich with documented cases, so it is worth pointing out that the phenomena controlling gas migration is not entirely specific to gases and could also apply to hydraulic fracturing fluid migration [21], which adds to the plausibility of aquifer contamination incidents.

Digiulio & Jackson [72] assessed the effect of fracturing operations on subsurface drinking water sources. The motivation behind this work was based on an EPA investigation done in 2008 in response to complaints in the Pavillion Field about changes in taste and odor of drinking water from domestic wells. To evaluate potential upward migration of fracturing fluid additives, the EPA drilled a couple of monitoring wells near the production wells (with a separating distance of 200–300 m) and sampled them. The two wells “MW01” and “MW02” were screened at intervals of 233–239 m and 296–302 m below ground surface, respectively. In the Pavillion Field, the maximum depth of groundwater use was 322 m (at the time the study was conducted) and some stimulation activities (acid stimulation/hydraulic fracturing) in the area occurred at depths as shallow as 213 and 322 m below ground surface. In the sampling results of the monitoring wells, several constituents of the fracturing fluid and/or their corresponding degradation products were detected, in considerable concentrations. Organic compounds that were detected include naphthalene, alkylbenzenes, diethylene glycol, octylphenol, and trimethylbenzene. These results provide extra credibility to the suggested scenario of the infiltration of fracturing fluid chemical additives into the drinking water sources.

It was possible for Digiulio & Jackson [72], via examination of previous incidents in the Pavillion field (not enough data is provided by the authors to document previous contamination incidents), to derive that the transport of additives of the fracturing fluid in the Pavillion field was possible due to the occurrence of the following phenomena: (i) casing failure facilitating seepage of the fluid, (ii) fracturing fluid injection into drinking water sources or near them, (iii) fluid leak-off via fractures, and (iv) lithological heterogeneity in the subsurface and the existence of permeable layers allowing fluid migration [32,92].

Based on facts and discussions analyzed in this section, it must be noted that even when the hydraulic fracturing fluid does not or takes several years to migrate towards the underground drinking water sources, it remains toxic posing a risk to human health, especially through the other existing exposure pathways. Human exposure to fracturing fluid chemicals primarily results from accidental spills and operational accidents. However, these chemicals are not exclusive to fracturing fluids; many are also components of other operational fluids or are, for example, hydrocarbon derivatives [78]. This overlap complicates the attribution of contamination solely to hydraulic fracturing, especially since detected chemicals in environmental samples sometimes consist of complex mixtures of organic compounds, like petroleum distillates [25,72,78]. Additionally, there exists a lack of robust pre-injection water quality checks in some regions, which complicates the ability to always demonstrate that the injection caused the contamination. So, to accurately pinpoint and limit contamination directly related to fracturing fluid injection, concentration of additives should not exceed the limits established by regulatory acts involved with monitoring the quality of drinking water quality [73]. In addition, not all the operators disclose fracturing fluid constituents they use, and the toxicity-related information is somewhat limited [19,20]. This obstructs investigations examining environmental hazards linked to the use of fracturing fluid.

The recognition and examination of environmental risks and health hazards resulting, during and/or after fracturing operations, from the usage of fracturing fluids takes several years [2]. As a result, long time periods must be allocated to track and simulate fracturing fluid transport in the subsurface [15]. Furthermore, low hydraulic fracturing fluid flow-back recovery values that are usually reported [[11], [12], [13]] prove that a relatively considerable fraction of the fluid is retained in the subsurface with the possibility of infiltrating into underground drinking water sources (e.g. Pavillion field). Consequently, the following section is dedicated to investigating fate of fracturing fluid and phenomena/parameters influencing its flow-back.

## 4 Fate of fracturing fluids

**Fig. 5:**
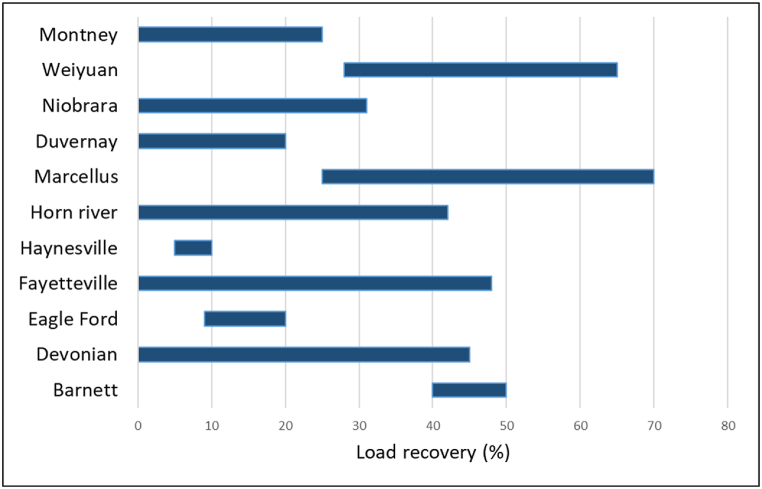
Load recovery values and ranges for shales (where accurate data was available, load recovery values were constrained in a range) (data obtained from Refs. [62,[93], [94], [95], [96]]).

Flow-back recovery of the fracturing fluid, termed as “load recovery”, is the fraction of the fracturing fluid that is recovered at the surface after the commencement of production. It is given in percentages and the first three months of production usually contribute to the main portion of the load recovery value [38]. According to reported values, load recovery can be relatively low and ranges between 10 and 50 % (Fig. 5).

The low recovery values are noteworthy since contaminants can migrate to the subsurface after production, and several factors control the fluid's migration and, consequently, its fate. This section discusses some of the key phenomena and points out the subsurface complexities that lead to impediments in studying fluid flow and assessing possible environmental impacts.

### 4.1 Fluid migration

Discrepancy in flow-back rates and ambiguity surrounding the fate of the fracturing fluid have garnered research interest due to the uncertainty in the causes [10,93] and the risk of aquifer contamination [14], which is strongly dictated by fluid migration and directly attributed to low flow-back ratios. The associated downhole risks have been previously addressed in sub-section 3.3.2. Low recovery values are the consequence of hydraulic communication resulting from the intersection between induced hydraulic fractures and permeable pathways previously existing in the formation, like faults and abandoned wells. There is a large number of published studies that investigated the upward flow of HF fluid and that gave, indirectly, insights regarding the fate of HF fluid that is not produced back [6,[21], [22], [23],[26], [27], [28],^97^,^98^]. For instance, Birdsell et al. [21] conducted a study where they simulated hydraulic fracturing fluid migration post injection into a Marcellus shale reservoir and the results showed that, over a period of 20 years, 68 % of the injected fracturing fluid was extracted by the production well, 0.1 % infiltrated into the overlying aquifer, and the remaining diffused elsewhere in the subsurface. The parameters that are positively correlated to, and control, the speed of fluid migration (presented from the most influential to least influential) are: 1) the proximity of a permeable pathway to HF, 2) permeable pathway permeability, 3) injection pressure, 4) permeable pathway width, 5) overburden heterogeneity, and 6) overburden permeability. As an illustration, the speed of fluid migration is higher in the case of higher proximity between HF region and the fault (i.e. shorter distance), when compared to cases of lower proximity [22,27,28,98].

### 4.2 Parameters affecting flow-back

#### 4.2.1 Correlations impacting load recovery

Correlations existing between load recovery and certain parameters (e.g. fracture network complexity, and shut-in period) have become a target of studies discussing fracturing fluid recovery, as illustrated in Table 4 which shows correlations derived specifically from numerical simulation studies. Negative correlation means that an increase in the ‘parameter studied’ yields a decrease in the load recovery, whereas a positive correlation means that an increase in the ‘parameter studied’ yields an increase in the load recovery.

**Table 4:** Correlations between formation and operational parameters and load recovery. Negative correlation means that an increase in the ‘parameter studied’ yields a decrease in the load recovery, whereas a positive correlation means that an increase in the ‘parameter studied’ yields an increase in the load recovery.

Parameter studied	Relation with load recovery	References
Shut-in period	Negatively correlated	[34]
		[38]
		[21]
		[35]
		[36]
		[37]
Natural fracture density	Negatively correlated	[36]
		[30]
		[5]
	Positively correlated	[99]
Fracture closure	Negatively correlated	[5]
		[99]
		[93]
	Positively correlated	[37]
Rock wettability	Hydrophobicity: positively correlated	[100]
		[101]
Hydraulic fracture permeability	Positively correlated	[21]
		[38]
		[30]
Reservoir pressure	Positively correlated	[100]
		[30]
Geochemical interactions	Negatively correlated	[10]
Fracture network complexity	Negatively correlated	[102]
		[35]
		[5]

Some of the correlations presented in Table 4 are expected. For example, a variety of studies have confirmed that extended shut-in periods and increased fracture network complexity promote hydraulic fracturing fluid imbibition into surrounding layers and fluid retention, respectively, leading to a reduction in load recovery. However, other parameters, such as natural fracture density, and fracture closure, exhibit intriguing correlations to load recovery.

#### 4.2.2 Effect of fracture network complexity

Literature findings support microseismic monitoring observations that demonstrate the development of complex fracture systems as a consequence of hydraulic fracturing [103]. Fracture network complexity in these systems arises from the existing natural fractures that are reactivated, and hydraulic fractures that are induced, and thus generates a broad spectrum of fracture dimensions. This complexity has its adverse effects on both fracturing fluid and hydrocarbon recovery rates [5,35,99].

Complex fracture networks developed through multi-stage hydraulic fracturing in tight reservoirs can contribute to low flow-back percentages due to fluid retention in the fracture network and fluid leak-off from the fracture to the matrix [5,29,30,32,93,102,104]. Fluid leakage phenomenon, known as “fluid loss”, is defined as the leakage of the fracturing fluid via the main fracture into the formation. Various numerical models dedicated to fluid leak-off simulation have been developed [[29], [30], [31], [32],^105^], and controlling properties were identified. Fluid leak-off is positively correlated to net fracture pressure, fracture length, natural fracture permeability and density, reservoir temperature and brine salinity. Conversely, reservoir pressure and fracturing fluid viscosity are negatively correlated with fluid leak-off [31,105].

Table 5 shows the load recovery values as a function of the fracture complexity index, as obtained by Ghanbari & Dehghanpour [35] and Liao et al. [5] in their respective simulation studies.

**Table 5:**
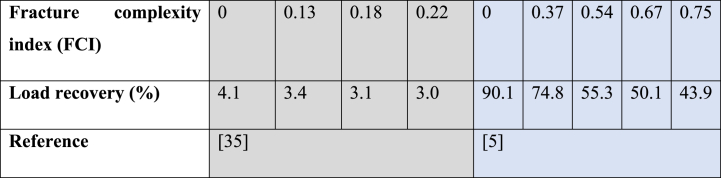
Load recovery results of two numerical studies as a function of fracture complexity.

Using flow-back data from 18 wells in the Horn River Basin, Ghanbari & Dehghanpour [35] categorized wells based on flow-back performance and gas production, linking higher fracture complexity to lower water recovery and higher gas production. To backup the basis of their categorization, Ghanbari & Dehghanpour [35] developed a numerical model of a flow-back process. To account for complexity in the model, a fracture complexity index (FCI) controlled by the volume of natural and hydraulic fractures was introduced and showed that increased fracture complexity restricts load recovery while promoting gas production. Liao et al. [5] employed the same FCI equation developed by Ghanbari & Dehghanpour [35] and derived similar conclusions. As anticipated, their results revealed that as the fracture network complexity increases, fracturing fluid load recovery declines. To illustrate, the load recovery decreased from 90 % to 43.9 % as the fracture complexity index increased from 0 to 0.75. However, despite demonstrating the same trend in the relation between fracture complexity and fluid recovery, there is a substantial disparity in load recovery values, with an order of magnitude difference. This discrepancy can be attributed to variations in values of model parameters, such as matrix permeability (0.01 mD in Liao et al.’s [5] study vs. 0.001 mD in Ghanbari & Dehghanpour's [35] study) and hydraulic fracture permeability (5000 mD in Liao et al.’s [5] study vs. 2000 mD in Ghanbari & Dehghanpour's [35] study).

Effect of fracture network complexity on fluids' flow and recovery can be explained as follows: During injection, the hydraulic fracturing fluid and proppants both fill the created hydraulic fracture while only the fluid flows into the natural fractures. Thus, the hydraulic fracture's aperture becomes greater than the natural fracture's aperture and the production of fluids from the hydraulic fracture becomes easier when compared to that from natural fractures. However, the higher the complexity of the fracture system created, the wider the contact surface area that is between the matrix and fractures. Hence, imbibition amplifies leading to an increase in the fraction of the water that gets imbibed into the shale matrix during shut-in, and displaces accumulated gas in fractures, which in-turn gets produced at higher ease by the production well. This leads to constrained load recovery and enhanced gas production during early production, and vice versa [35].

##### 4.2.2.1 Effect of natural fracture density

Natural fracture density is one of the fracture network characteristics that determines the degree of complexity. The findings for the numerical simulations by Liu et al. [99] contradict other models' [5,30,36] that proposed that the increased contact area due to an increase in natural fracture density results in the retention of higher volumes of water in the subsurface. Liu et al.’s [99] results may be considered as a demonstration of the counter-effect of an increased contact area, which could yield a boost in hydraulic communication and hence in water recovery. The apparent contradiction might be due to the different scales and input of these models. For example, natural fracture permeability in Liao et al.’s [5] study is three orders of magnitude higher than the value used in Liu et al.’s [99] study.

##### 4.2.2.2 Effect of fracture closure

Another noteworthy feature is the fracture closure. Fracture closure hinders the flow of a fraction of the fracturing fluid from the fractures back to the well, thereby increasing retained volume [93]. While some studies support that accounting for fracture compressibility variations leads to lower load recovery [5,99], the study done by Seales et al. [37] shows that fracture closure mechanisms might actually “squeeze” the water out of the saturated fractures and enhance early flow-back. This divergence in findings could stem from differing modeling assumptions. In the studies by Liu et al. [99] and Liao et al. [5], fracture permeability varied with factors like fracture closure and pressure changes, whereas Seales et al.’s [37] model assumed it to be constant.

In conclusion, while fracture network complexity generally leads to lower fluid recovery and higher hydrocarbon production, the specific outcomes depend on a range of factors, including fracture closure properties and the interactions between natural and induced fractures. These complexities highlight the challenges in accurately predicting well performance and fluid flow-back. Additionally, in complex geological settings with local heterogeneities, in-situ stress fields, spatial inconsistency in rock mass properties, and pre-existing bedding planes and fault systems, hydraulic fracturing operations become more challenging. These factors, coupled with the complexities in fracture network characterization, highlight the difficulties in accurately predicting flow-back and hydrocarbon recovery [99,106,107].

## 5 Induced seismicity and load recovery correlation

In studies examining seismicity referred to hydraulic fracturing activities, the primary focus is often on seismic events arising from wastewater injection [108,109]. This process involves the disposal of flowback water, which consists of the original fracturing fluid (i.e. water and chemical additives) along with naturally present constituents from the formation, into designated wells [87,110]. This emphasis is due to the fact that earthquakes triggered by wastewater injection generally exhibit higher magnitudes than those directly induced by hydraulic fracturing, with recorded events reaching magnitudes of 5.6 [111], 5.8 [70,87], and even 5.9 [112]. These elevated magnitudes are typically ascribed to the substantial volumes of injected wastewater and the resultant pore pressure increases, which can stimulate seismic activity [87,109]. Nonetheless, induced earthquakes recorded in areas near hydraulic fracturing sites can be attributed to hydraulic fracturing processes [[39], [40], [41], [42],^44^,^45^,^87^,[111], [112], [113], [114], [115], [116], [117], [118]], especially under specific hydrogeological conditions [42,119,120]. A spatial and temporal link has been developed in regions like the Western Canada Sedimentary Basin (WCSB) [[39], [40], [41]] and the Sichuan Basin, China [43,44]. This association is supported by data from well monitoring and the compilation of operational data and seismic activity into catalogs [39,43,44,114,116,118]. An example of such data is the one compiled by Verdon & Rodriguez-Pradilla [114] (see Table 6), showcasing seismic activity related to fracturing in 12 major shale plays in the Western Canada Sedimentary Basin (WCSB) and the United States using data from regulators (i.e., Alberta Energy Regulator and the British Columbia Oil and Gas Commission) and FracFocus (www.fracfocus.org), respectively.

**Table 6:** Hydraulic fracturing-induced seismic activity in 12 major shale plays (adapted from [114]).

Play	Number of wells	Total HF volume (x 10^6^ m^3^)	Number of associated earthquakes	Largest event magnitude	Number of associated wells
Bakken	15,375	288	1	3.3	3
Barnett	6427	45	2	2.9	3
Duvernay	1946	44	569	4.1	145
Eagle Ford	15,825	407	389	3.7	731
Fayetteville	2863	28	421	3.9	336
Haynesville	3650	208	18	3.8	9
Horn River	344	14	30	3.6	25
Marcellus	11,530	456	2	2.8	2
Montney	8281	80	388	4.4	603
Niobrara	17,314	367	9	2.6	45
Utica	3146	157	37	3.8	24
Woodford	4424	198	794	3.6	584

Although no formula can be derived to link number of wells or any other parameter to the magnitude or occurrence of seismic events, this kind of data is highly useful for studying the relationship between hydraulic fracturing and induced seismicity. Verdon & Rodriguez-Pradilla [114] specifically acknowledge that not all earthquakes are caused by hydraulic fracturing, as some may result from natural causes or other industrial activities. To improve data accuracy and ensure that the majority of the listed events are induced by hydraulic fracturing, they applied the following criteria:

(i) Used a 10 km distance from the operation site.
(ii) Counted seismic events with magnitude ≥2.0 from the beginning of operations to 10 days after they concluded.
(iii) Compared seismic activity during operations to the baseline activity observed in the area 6 months before and 6 months after the operational period.

One of the fundamental processes responsible for fluid induced seismicity is determined to be fluid diffusion, succeeding fluid injection. This diffusion is enabled due to the hydraulic communication established between hydraulic fractures and faults, allowing the fracturing fluid to reach existing faults. Hence, pre-existing faults are reactivated due to pore pressure increase during or after hydraulic fracturing that is enough to trigger earthquakes [42,44,113]. Fluid pressurization and injection have become key research attractions due to their role in fault reactivation [45,113]. He & Li [113] used the data presented in Table 7 and conducted a simulation study that demonstrated that amplified fluid injection increases the probability of fault reactivation. As per the data presented by Wu et al. [45] in their review paper (see Table 7), we can highlight that fluid injection in any unconventional energy project has the potential to induce seismicity, which broadens the scope of research concerned with the injection and fate of the fracturing fluid.

**Table 7:** Data showing the link between flow back and HF and induced seismicity.

Reference	Presented Data		
[115]	**Treatment stage**	**Flow-back after the stage**	**Seismicity associated with the stage**
	II	No flow-back	First seismic event
	III	Aggressive flow-back	Weak seismicity
	IV	No flow-back	Significant seismicity
	V	Aggressive flow-back	Weak seismicity
	Overall flow-back	20 %	
[45]	**Location**	**Reason**	**Significant recorded magnitude**
	Pohang, South Korea	Geothermal reservoir stimulation	MW = 5.5
	Sichuan, China	Shale gas production	MW = 4.7
	Oklahoma, United States	Wastewater disposal	MW > 4.0
[113]	**Data**		**Value**
	Five earthquakes recorded during fracturing		MW > 4.0
	Magnitude of largest recorded event		MW = 4.7
	Distance separating the source of seismicity and injection point		2–3 km
[39]	Magnitude of largest recorded event		MW = 3.9
	Time of occurrence of largest recorded event		Two weeks after fracturing completion
	Distance separating clusters from fractured wells		2 km
	Flow-back		7 %
[44]	Period between start of HF and first recorded seismic event		5 days
	Period between start of HF and largest recorded seismic event		3 months
	Magnitude of largest recorded event		MW = 2.2
	Flow-back		40 %

According to Schultz et al. [41], the injected fluid volume is the dominating operational parameter that is directly positively correlated to both the number of seismic events recorded and their magnitude. Although this behavior is expected, a fluid injection over a long period also boosts fluid seepage into surrounding formation [118], which in turn affects flow-back recovery. In their report done to study induced seismicity in the Bowland Shale in the UK, De Pater & Baisch [115] represented a plausible relation between fracturing flow-back and induced seismicity. Table 7 illustrates the degree of seismicity associated with each stage of hydraulic fracturing as a function of the well flow-back. They suggested that the pattern of seismicity shown is the result of flow-back routines, where strong seismicity is associated with low fluid flow-back volumes, and the fact that only about 20 % of the injected fluid was recovered in this operation supports their observations. Hence, they recommended aggressive fluid flow-back and reduced injected volumes as possible seismicity mitigation practices. Likewise, Bao & Eaton [39] compiled data of a four-month period for six hydraulic fracturing well pads in the Duvernay formation, and the most interesting data are presented in Table 7. Moreover, Tan et al. [44] used available injection data from two well pads, named “N5” and “N7”, in Sichuan Basin, as a basis for their study. Data derived from pad N5 shows the time period separating the onset of fracturing activities and seismic events occurrence (Table 7), and the flow-back ratio. Values synthesized in Table 7 prove the suggested link between hydraulic fracturing and induced seismicity as per the discussion above, and provide evidence that the earthquake distributions are spatially concentrated nearby hydraulic fracturing well pads and follow chronologically the timing of stimulation activities by few days or weeks.

Although conclusions discussed in this section might be considered redundant, they act as support to the hypothesis that connects induced seismicity to low fracturing fluid flow-back recovery since authors present fluid diffusion as one of the major phenomena contributing to induced seismicity in the proximity of hydraulic fracturing locations. For instance, Tan et al. [44] and Bao & Eaton [39] reported load recovery values in cases where fluid diffusion was believed to be the main trigger behind seismic events (i.e., earthquakes), and these values show that more than 50 % and 90 %, respectively, of the injected fluid remained trapped in the subsurface in these cases. Nevertheless, it is worth highlighting that the magnitude of the events is a result of a combination of factors including the injection pressure, cumulative injected volumes, position of created fractures with respect to existing faults, and hydraulic communication between the fractures and these faults which is also discussed in Yehya et al. [42].

## 6 Emerging technologies in fracturing fluids

Recent advancements in hydraulic fracturing technology aim to develop environmentally friendly fluids to reduce toxicity and environmental impact [[121], [122], [123]], while also addressing technical challenges associated with conventional fracturing fluids [55,121,122,124]. This shift involves the transition from traditional fluids, such as water- and oil-based systems, to unconventional alternatives like viscoelastic surfactant (VES) fluids, liquefied petroleum gas (LPG)-based fluids, and CO₂-based fluids [56]. These new formulations aim to minimize issues like permeability reduction, pore clogging, and formation damage caused by residues left by polymer-based fracturing fluids [87,122].

In particular, viscoelastic surfactant (VES) fluids are gaining attention for their capacity to act as clean fluids, leaving minimal residue and providing enhanced environmental performance [55,56,87,122,125,126]. VES fluids address common limitations of polymer-based fluids by eliminating insoluble residues, reducing friction, and limiting formation damage [55,122].

To further improve environmental safety and performance, researchers are exploring the use of alternative additives in these fluids. These include biodegradable and green surfactants, nanocomposite friction reducers, hydrogel additives, and amino acid-based surfactants, each offering enhanced biodegradability and lower ecological impact [87,121,124]. By incorporating such components, fracturing fluids are engineered to deliver higher lubricity, increased temperature resistance, and better proppant transport, thus supporting efficient hydraulic fracturing with reduced environmental risks [87,121,122].

Overall, these innovations towards green alternatives for fracturing fluid reflect a growing emphasis on sustainability in hydraulic fracturing, balancing improved operational performance with reduced environmental footprint. However, parallel to this progress, equal efforts should be dedicated to evaluating and addressing potential environmental risks associated with these emerging alternatives.

## 7 Conclusions and outlook

The public, in general, and environmentalists, in specific, are consistently raising concerns regarding risks associated with the rapid expansion of hydraulic fracturing treatments worldwide. Hydraulic fracturing fluid poses environmental risks that can be classified into surface risks (e.g. spills, felt seismic events), and subsurface risks (e.g. groundwater contamination). Also, more than half of the injected volume of fracturing fluid is not produced back to the surface, and the higher the percentage of fracturing fluid that remains in the subsurface, the higher are the risks associated with it.

In this work, the main objective is not to discuss the risks related to all the stages of a hydraulic fracturing job, but to discuss the environmental impact of the fracturing fluid. First, we discuss the environmental and health risks associated with HF fluid and its implications on drinking water and highlight reported cases of contamination. Then, we inspect available field data on flow-back recovery rates and load recoveries of the HF fluid, and summarize the parameters and hydrogeological conditions controlling the values of recovery. Hence, we show how HF fluid recovery correlates with the fluid's upward migration through permeable pathways, retention in fracture networks, leak-off into the formation, and induced seismicity.

Main conclusions derived from the literature can be summarized as follows:

1. The high number of toxic chemicals added to the HF fluid and the limited disclosure of toxicity data highlight the importance of addressing the surface and subsurface risks of HF fluid.
2. Load recovery is correlated to fracture properties (e.g. natural fracture density) and operational parameters such as shut-in period. Most evidently long shut-in period leads to low hydraulic fluid recovery values.
3. Fluid migration in the subsurface via a permeable pathway, which contributes to low recovery and aquifer contamination, is mostly sensitive to properties of the existing permeable pathway. For instance, a strong positive correlation exists between speed of fluid migration and permeability of the pathway.
4. Fluid leak-off from the fracture to the matrix is considered to be a strong contributor to the low load recovery values due to the retention of the HF fluid.
5. Higher fracture network complexity boosts oil/gas recovery and curbs water recovery.
6. Field evidence support the hypothesis suggesting a negative correlation between induced seismicity in the vicinity of fracturing operations and load recovery; which is explained by fluid diffusion into fractured regions.

Environmental impact of the HF fluid content and fate (e.g., migration) of the HF fluid are interrelated and must be evaluated simultaneously to assess related risks, and therefore be able to make decisions regarding hydraulic fracturing operations’ design. Accordingly, when assessing environmental risks, it is essential to examine the underlying causes of low recovery rates and the parameters and phenomena that influence fluid transport and recovery. Recent literature stresses on the need for a comprehensive investigation into the causes of increased seismicity proximal to hydraulic fracturing sites, as hydraulic fracturing is employed not only in shale development but also in renewable energy projects.

Further research is essential to specifically address the environmental impacts of fracturing fluids as complex chemical mixtures, independent of the hydraulic fracturing processes. Although existing studies provide insight into potential environmental risks, the majority concentrate on the operational aspects of hydraulic fracturing rather than the toxicity and long-term environmental effects of the fluid's chemical constituents. Comprehensive risk assessment requires more extensive chemical analysis of fracturing fluids, as well as toxicological and epidemiological data that quantify exposure risks to humans and animals. Also, detailed exposure assessments are necessary to understand the pathways through which these chemicals affect biological systems. This will assist in the development of appropriate safety precautions and disclosure mandates that reduce the harm.

Furthermore, implications on groundwater contamination might take tens of years to be detected, so, serious efforts should be put in place to gain more access to data needed to develop risk mitigation plans that take into consideration the timescales associated with the conditions and mechanisms of fluid migration in the subsurface.

Finally, while innovative approaches to create more sustainable fracturing fluid alternatives are promising, equal attention must be dedicated to a thorough evaluation of their environmental impacts to ensure they meet both safety and sustainability standards while achieving the desired fracturing outcomes.

## CRediT authorship contribution statement

**Sara Makki:** Writing – original draft, Methodology, Investigation, Formal analysis, Data curation. **Elsa Maalouf:** Writing – review & editing, Supervision, Methodology, Funding acquisition, Formal analysis, Conceptualization. **Alissar Yehya:** Writing – review & editing, Supervision, Methodology, Funding acquisition, Formal analysis, Conceptualization.

## Data availability statement

The data that support the findings of this study are available on request from the corresponding author.

## Funding statement

This research is funded by the University Research Board at the 10.13039/100007688American University of Beirut, Awards #104392 and #104260.

## Declaration of competing interest

The authors declare that they have no known competing financial interests or personal relationships that could have appeared to influence the work reported in this paper.
